# J-shaped association of dietary catechins intake with the prevalence of osteoarthritis and moderating effect of physical activity: an American population-based cohort study

**DOI:** 10.3389/fimmu.2023.1287856

**Published:** 2024-01-08

**Authors:** Yuesong Fu, Lu Li, Jing Gao, Fazheng Wang, Zihan Zhou, Yiwei Zhang

**Affiliations:** ^1^ Department of Orthopedics, The First People’s Hospital of Kashi Prefecture, Kashi, Xinjiang, China; ^2^ Department of Hand Surgery and Peripheral Neurosurgery, The First Affiliated Hospital of Wenzhou Medical University, Wenzhou, Zhejiang, China; ^3^ Department of Orthopedics, Shanghai Tenth People’s Hospital, Tongji University School of Medicine, Shanghai, China

**Keywords:** osteoarthritis, catechins, physical activity, epigallocatechin, epigallocatechin 3-gallate

## Abstract

**Background:**

Catechins are a class of natural compounds with a variety of health benefits, The relationship between catechins and the prevalence of osteoarthritis (OA) is unknown. This study investigated the associations between daily intake of catechins and the prevalence of OA among American adults and assessed the moderating effect of physical activity (PA).

**Methods:**

This study included 10,039 participants from the National Health and Nutrition Examination Survey (2007–2010,2017-2018). The logistic regression, weighted quantile sum (WQS) regression, and restricted cubic spline (RCS) regression models were conducted to explore the associations between daily intake of catechins and the prevalence of OA. Moreover, interaction tests were performed to assess the moderating effect of PA.

**Results:**

After multivariable adjustment, the weighted multivariable logistic regression and RCS regression analyses revealed significant J-shaped non-linear correlations between intakes of epigallocatechin and epigallocatechin 3-gallate had significant associations with the prevalence of OA among in U.S. adults. WQS regression analysis showed that excessive epigallocatechin intake was the most significant risk factor for OA among all subtypes of catechins. In the interaction assay, PA showed a significant moderating effect in the relationship between epigallocatechin intake and OA prevalence.

**Conclusions:**

The intake of gallocatechin and gallocatechin 3-gallate had a significant negative correlation with the prevalence of OA and the dose-response relationship was J-shaped.PA below 150 MET-min/week and the threshold intakes of 32.70mg/d for epigallocatechin and 76.24mg/d for epigallocatechin 3-gallate might be the targets for interventions to reduce the risk of developing OA.

## Introduction

Osteoarthritis (OA) is a degenerative and inflammatory joint disease caused by multiple factors, which is characterized by joint swelling, pain and cartilage destruction (1, 2). Although the underlying mechanism is not fully understood, its impact on the quality of life of patients is beyond doubt. According to epidemiological statistics, the age-standardized prevalence rate of OA in the United States increased by 23.2% from 1999-2017, which is one of the highest age-standardized prevalence rate increases of OA in the world (3). It is estimated that the number of OA patients in the USA will increase to 67 million by 2030 (4). The financial expenditure on OA care is estimated at US $15.5-28.6 billion per year (2). OA is becoming a disease that attracts more and more attention.

Flavonoids are a group of polyphenolic compounds derived from plant secondary metabolism. They are widely present in a variety of foods, such as vegetables, fruits, tea and wine (5, 6). According to their chemical structure, they can be divided into six subgroups, namely flavonoids, flavanones, flavonols, isoflavones, anthocyanins and flavanols (7–11). Catechins are a subgroup of flavonols. There were eight monomers of catechins, catechin (C), epicatechin (EC),gallocatechin (GC), epigallocatechin (EGC), catechin gallate (CG), epicatechin gallate (ECG), gallocatechin gallate (GCG) and epigallocatechin gallate (EGCG) (12–14). In recent years, with the deepening of research, catechins have been proven to have a kind of biological activities such as anti-inflammatory, anti-oxidation, anti-cancer and bone protection (6, 12, 15, 16). Therefore, supplemental catechins are increasingly recognized as nutritional supplements for the treatment of many diseases, including OA.

The relationship between physical activity and OA has been well-paid attention to in recent years. Homeostasis of joints and joint damage are regarded as the main causes of OA caused by PA (17–20). In addition, the loss of muscle strength caused by low-intensity PA is also the key to the elderly getting OA (21). However, for the general population, daily PA does not increase the risk of joint OA, and moderate levels of PA can also improve soft tissue ductility, blood flow, and synovial fluid mobility, maintain normal joint range of motion, and provide essential nutrients to the cartilage matrix (18, 22, 23).

Since catechins are easily accessible in daily life and have shown excellent medical effects, the present study, which used the NHANES database, attempted to investigate the relationship between daily intake of catechins and the prevalence of OA and to explore whether physical activity plays a moderating role.

## Methods

### Study population

The National Health and Nutrition Examination Survey (NHANES) is a periodic cross-sectional random sample survey of the non-institutional population in the United States. Survey data are compiled by professionals and released on the NHANES official website for public access (https://www.cdc.gov/nchs/nhanes/). NHANES was approved by the Division of Health and Nutrition Examination Surveys (DHANES) and the National Center for Health Statistics (NCHS), and all participants provided written informed consent.

This study included all 29,940 participants in the “2007-2008”, “2009-2010” and “2017-2018” survey cycles, which have published data on dietary flavonoid intake. After excluding individuals with missing osteoarthritis, catechins, and covariates data, a total of 10039 participants were included in this study.

### OA status

Participants with OA were defined by previous physician diagnosis, and collected by trained interviewers through questionnaires. Shortly, participants aged ≥20 years were asked two questions related to arthritis: “Has a doctor or other health professional ever told you that you have arthritis?” and “Which type of arthritis was it?” Participants who responded “yes” and “osteoarthritis” were divided into the osteoarthritis group and the other participants were divided into the Non-osteoarthritis group.

### Dietary catechins intake assessment

Catechins are widely found in plants and can be consumed in the daily diet through fruits, tea, coffee, and so on (24). Dietary catechins intake data were extracted from the Food and Nutrient Database for Dietary Studies (FNDDS), which used 24-hour dietary recall interview data from NHANES to calculate participants’ nutrient intakes over 24 hours. In FNDDS, flavonoids were subdivided into 29 species, of which (-)-Epicatechin, (-)-Epicatechin 3-gallate, (-)-Epigallocatechin, (-)-Epigallocatechin 3-gallate, (+)-Catechin, (+)-Gallocatechin were included in the “total catechins” category.

### Physical activity assessment

Physical activity (PA) data were collected by trained interviewers through a global physical activity questionnaire survey. The metabolic equivalent (MET) of weekly PA was calculated by multiplying the weekly minutes of moderate or vigorous PA, frequency, and MET value recommended by NHANES. According to the WHO guidelines, participants who achieved moderate intensity (150 min per week) were categorized into the sufficiently PA group (>600 MET-min/wk) and the rest into the insufficiently PA group (600-150 MET-min/wk), low PA group (<150 MET-min/wk), and inactive PA group (PA=0) (25).

### Covariates

Demographic information, including age, gender, ethnicity, education level, marital status, and poverty income ratio (PIR), was collected by trained interviewers using a questionnaire standardized. History of hypertension and diabetes were determined by the use of prescribed medications and previous physician diagnosis. Body mass index (BMI) was measured by professionals in the Mobile Examination Center (MEC). Defined as a drinker based on ≥ 12 drinks per year and < 12 drinks per year as a non-drinker. Smokers were defined as having smoked more than 100 cigarettes in the past.

### Statistical analyses

Considering the complex sampling design of NHANES, the dietary day one sample weights were included in all analyses of this study. In the characterization of osteoarthritis and non-osteoarthritis participants, normally distributed continuous variables were presented as mean (standard deviation), catechins intake was presented as median (25th, 75th) due to non-normal distribution, and categorical variables were expressed as absolute values (weighted percentages). Statistical differences between the two groups were tested by t-tests, Wilcoxon rank sum tests, and Rao-Scott Chi-square tests., respectively. Univariate and multivariate logistic regression analyses adjusting for the confounding factors and trend tests for the Q group of catechins were used to investigate the 6 catechins and total catechins intake in relation to the prevalence of OA. Model 1 was a univariate logistic regression model without adjustment. Model 2 was the multivariate logistic regression model adjusted for age (20–59 years, ≥60 years), gender (male, female), and ethnicity (non-Hispanic White, non-Hispanic Black, Mexican American, Other race). Model 3 was additionally adjusted for education level (less than high school, high school or equivalent, and college or above), marital status (married/cohabiting, widowed/divorced/separated, never married), PIR, BMI, smoke, alcohol drinking, and history of diabetes or hypertension. The restricted cubic spline (RCS) regression model was performed to investigate the non-linear relationship between the intake of Epigallocatechin and Epigallocatechin 3 gallate with the risk of OA. The number of knots for the RCS regression was three, with the smallest Akaike information criterion to ensure the best fit. Weighted quantile sum (WQS) regression models were performed to evaluate the relationship between indexes representing six catechins or Epigallocatechin and Epigallocatechin 3 gallate co-exposure and the risk of OA. The likelihood ratio test was used to test the significance of the effect on the risk of OA caused by the multiplicative interaction term of PA with Epigallocatechin or Epigallocatechin 3 gallate. Multiple sensitivity analyses are conducted to check the robustness of our results, including logistic regression models adjusting for different covariates, weighted regression models, stratified analyses, and interaction tests. All analyses were conducted using R software (version 4.2.1), and a bilateral *P* value less than 0.05 was considered statistically significant.

## Result

### Basic characteristics and catechins intake of study participants

As shown in **Table 1**, a total of 10,039 volunteers from the NHANES were enrolled in our study, including 8839 non-osteoarthritis and 1,200 osteoarthritis patients. Interestingly, in addition to catechin and gallocatechin, we found that intakes of epigallocatechin, epigallocatechin 3-gallate, epicatechin, epicatechin 3-gallate, and total Catechins were statistically different between the OA and non-OA populations (P < 0.05). In addition, there were significant differences in age, gender, ethnicity, marital status, BMI, poverty income ratio, smoke, diabetes, hypertension, and physical activity between the OA and non-OA populations (P < 0.05). Among them, those aged ≥60 years old, female, divorced, widowed, obesity, smoke, diabetes, and hypertension were more likely to have osteoarthritis.

**Table 1:** Characteristics and catechins intake in participants stratified by OA in the US (NHANES 2007-2010 and 2017-2018).

Characteristic	All participants (10039)	Osteoarthritis (1200)	Non- Osteoarthritis^a^ (8839)	*p* value^b^
**Age (years), n (%)**				< 0.0001
20-59	7170 (71.42)	393 (42.38)	6777 (84.38)	
> = 60	2869 (28.58)	807 (57.62)	2062 (15.62)	
**Gender, n (%)**				< 0.0001
Female	4969 (49.5)	773 (65.41)	4196 (48.80)	
Male	5070 (50.5)	427 (34.59)	4643 (51.20)	
**Ethnicity, n (%)**				< 0.0001
Non-Hispanic Black	1891 (18.84)	170 (5.88)	1721 (10.85)	
Non-Hispanic White	4614 (45.96)	784 (82.96)	3830 (66.64)	
Mexican American	1684 (16.77)	96 (3.02)	1588 (9.36)	
Other Hispanic	973 (9.69)	79 (2.32)	894 (5.74)	
Other Race	877 (8.74)	71 (5.82)	806 (7.40)	
**Education level, n (%)**				0.76
Less than high school	2333 (23.24)	245 (13.29)	2088 (14.04)	
High school or equivalent	2333 (23.24)	288 (25.58)	2045 (24.62)	
College or above	5373 (53.52)	667 (61.13)	4706 (61.34)	
**Marital status, n (%)**				< 0.0001
Married/cohabiting	6102 (60.78)	712 (64.90)	5390 (61.90)	
Widowed/divorced/separated	2031 (20.23)	418 (28.05)	1613 (15.23)	
Never married	1906 (18.99)	70 (7.05)	1836 (22.87)	
**Poverty income ratio**	3.13 (0.04)	3.10 (0.04)	3.34 (0.09)	0.01
**Body mass index (kg/m2)**	28.73 (0.12)	28.49 (0.13)	30.42 (0.32)	< 0.0001
**Smoke, n (%)**				< 0.001
no	5665 (56.43)	573 (49.99)	5092 (58.48)	
yes	4374 (43.57)	627 (50.01)	3747 (41.52)	
**Alcohol drinking, n (%)**				0.28
Non-drinker	1249 (12.44)	162 (10.57)	1087 (9.29)	
Drinker	8790 (87.56)	1038 (89.43)	7752 (90.71)	
**Diabetes, n (%)**				< 0.0001
no	8957 (89.22)	965 (84.36)	7992 (93.43)	
yes	1082 (10.78)	235 (15.64)	847 (6.57)	
**Hypertension, n (%)**				< 0.0001
no	6188 (61.64)	394 (39.38)	5794 (70.60)	
yes	3851 (38.36)	806 (60.62)	3045 (29.40)	
**Physical activity, n (%)**				< 0.0001
Inactive	2435 (24.26)	2024 (18.42)	411 (26.27)	
Low	1061 (10.57)	913 (10.08)	148 (12.68)	
Insufficiently	176 (1.75)	138 (1.53)	38 (3.82)	
Sufficiently	6367 (63.42)	5764 (69.97)	603 (57.22)	
**Catechin (mg/day)**	4.99 (1.13, 11.03)	6.05 (1.49, 12.21)	4.84 (1.08, 10.87)	0.05
**Gallocatechin (mg/day)**	0.00 (0.00, 1.08)	0.01 (0.00, 1.08)	0.00 (0.00, 1.08)	0.21
**Epicatechin (mg/day)**	4.74 (0.79, 15.33)	7.05 (1.42, 16.28)	4.46 (0.72, 15.20)	0.003
**Epicatechin 3-gallate (mg/day)**	0.01 (0.00, 1.38)	0.02 (0.00, 10.38)	0.00 (0.00, 1.03)	0.001
**Epigallocatechin (mg/day)**	0.40 (0.06, 3.73)	0.73 (0.20, 16.30)	0.35 (0.04, 3.06)	< 0.0001
**Epigallocatechin 3-gallate (mg/day)**	0.00 (0.00, 3.37)	0.11 (0.00, 24.34)	0.00 (0.00, 2.76)	< 0.001
**Total Catechins (mg)**	12.41 (3.19, 54.31)	16.38 (4.95, 79.50)	12.01 (3.04, 52.35)	0.004

a The non-osteoarthritis group was defined as participants who had not self-reported any kind of arthritis.

b Statistical differences between the two groups were tested by t-tests for normally distributed continuous variables, Wilcoxon rank sum tests for non-normal distributed continuous variables, and Rao-Scott Chi-square tests for categorical variables.

### The associations between six catechins subclass intakes and the prevalence of OA

**Figure 1:**
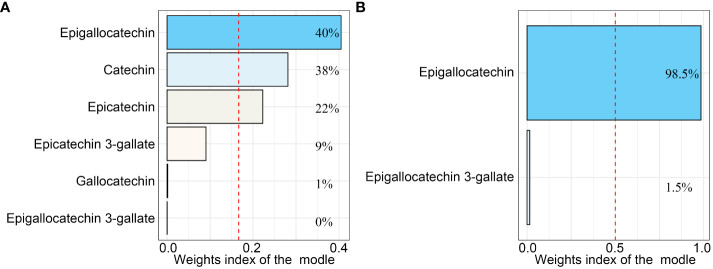
The weighted quantile sum (WQS) regression model index weights for the WQS regression model that included **(A)** all catechins and **(B)** only Epigallocatechin and Epigallocatechin 3-gallate. The red dashed line indicates the inverse of the number of exposed variables in the model. All the models were adjusted for age, gender, ethnicity, education level, marital status, poverty income ratio, body mass index, serum cotinine, alcohol drinking status, and history of diabetes or hypertension.

Firstly, we used logistic regression models to analyze the single effect of each catechin subclass on the prevalence of OA (**Table 2**). Interestingly, we found, in model 3 adjusted for all covariates, intakes of epigallocatechin (P for trend = 0.02) and epigallocatechin 3-gallate (P for trend = 0.01) showed significant associations with OA in the third Qs. Compared to the Q 1 group, epigallocatechin (OR: 1.76, 95% CI: 1.28, 2.42) and epigallocatechin 3-gallate (OR: 1.338, 95% CI: 1.08, 1.77) in the Q 3 group presented a higher risk of OA. In addition, we further used the WQS regression model to analyze the mixed effects of total catechins subclass intakes on the risk of OA (**Table 3**), and the results showed that the WQS index had no statistical significance with OA risk reduction. Subsequently, we selected epigallocatechin and epigallocatechin 3-gallate for re-analysis, and the results showed that the WQS index was significantly correlated with increased OA risk (OR: 1.04, 95% CI: 1.00,1.08). The estimated weights of epigallocatechin and epigallocatechin 3-gallate in the WQS regression model are shown in **Figure 1B**.

**Table 2:** The association between the catechins intake and osteoarthritis in the US (NHANES 2007-2010 and 2017-2018).

Variable	Q 1	Q 2		Q 3		*p* for trend
Catechin (mg/day)						
Model 1 [OR (95% CI), *p*]	Reference	1.15 (0.92, 1.43)	0.22	1.25 (0.95, 1.66)	0.11	0.15
Model 2 [OR (95% CI), *p*]	Reference	0.81 (0.66, 0.99)	0.95	1.09 (0.80, 1.50)	0.56	0.52
Model 3 [OR (95% CI), *p*]	Reference	1.10 (0.85, 1.42)	0.46	1.26 (0.90, 1.77)	0.18	0.18
Gallocatechin (mg/day)						
Model 1 [OR (95% CI), *p*]	Reference	1.78 (1.36, 2.31)	< 0.0001	1.07 (0.85, 1.34)	0.57	0.50
Model 2 [OR (95% CI), *p*]	Reference	1.40 (1.03, 1.90)	0.03	1.06 (0.81, 1.40)	0.65	0.86
Model 3 [OR (95% CI), *p*]	Reference	1.56 (1.11, 2.18)	0.01	1.12 (0.85, 1.47)	0.40	0.99
Epicatechin (mg/day)						
Model 1 [OR (95% CI), *p*]	Reference	1.47 (1.15, 1.88)	0.003	1.45 (1.06, 1.97)	0.02	0.13
Model 2 [OR (95% CI), *p*]	Reference	1.25 (0.96, 1.62)	0.09	1.21 (0.86, 1.72)	0.27	0.54
Model 3 [OR (95% CI), *p*]	Reference	1.36 (1.03, 1.80)	0.03	1.35 (0.93, 1.94)	0.11	0.31
Epicatechin 3 gallate (mg/day)						
Model 1 [OR (95% CI), *p*]	Reference	1.50 (1.15, 1.94)	0.003	1.53 (1.22, 1.92)	< 0.001	0.001
Model 2 [OR (95% CI), *p*]	Reference	1.19 (0.88, 1.60)	0.25	1.18 (0.91, 1.53)	0.20	0.29
Model 3 [OR (95% CI), *p*]	Reference	1.30 (0.93, 1.80)	0.12	1.28 (0.96, 1.72)	0.09	0.16
Epigallocatechin (mg/day)						
Model 1 [OR (95% CI), *p*]	Reference	2.07 (1.69, 2.54)	< 0.0001	2.55 (1.95, 3.33)	< 0.0001	< 0.0001
Model 2 [OR (95% CI), *p*]	Reference	1.40 (1.11, 1.78)	0.01	1.68 (1.25, 2.25)	< 0.001	0.01
Model 3 [OR (95% CI), *p*]	Reference	1.37 (1.07, 1.77)	0.02	1.76 (1.28, 2.42)	< 0.001	0.02
Epigallocatechin 3 gallate (mg/day)						
Model 1 [OR (95% CI), *p*]	Reference	1.52 (1.16, 1.99)	0.003	1.60 (1.32, 1.94)	< 0.0001	< 0.0001
Model 2 [OR (95% CI), *p*]	Reference	1.17 (0.82, 1.67)	0.37	1.28 (1.02, 1.62)	0.03	0.03
Model 3 [OR (95% CI), *p*]	Reference	1.31 (0.91, 1.88)	0.14	1.38 (1.08, 1.77)	0.01	0.01
Total catechins (mg/day)						
Model 1 [OR (95% CI), *p*]	Reference	1.31 (1.05, 1.63)	0.02	1.41 (1.06, 1.87)	0.02	0.08
Model 2 [OR (95% CI), *p*]	Reference	1.19 (0.93, 1.52)	0.17	1.17 (0.85, 1.61)	0.33	0.59
Model 3 [OR (95% CI), *p*]	Reference	1.30 (1.00, 1.70)	0.05	1.31 (0.93, 1.85)	0.12	0.31

Model 1: No adjustment. Model 1: Adjusted for age, gender, ethnicity. Model 2: Adjusted for age, gender, ethnicity, education level, marital status, poverty income ratio, body mass index, smoke, alcohol drinking status, and history of diabetes or hypertension. CI, confidence interval; OR, odds ratio.

**Table 3:** The association between the catechins intake and osteoarthritis in the US by WQS analysis in the US (NHANES 2007-2010 and 2017-2018).

	Adjusted OR (95% CI)	*p*-value
WQS (all catechins)^a^	1.03 (0.99, 1.07)	0.11
WQS (Epigallocatechin and Epigallocatechin 3-gallate)^b^	1.04 (1.00, 1.08)	0.04

a The WQS regression model containing all catechins as exposure factors.

b The WQS regression model containing epigallocatechin and epigallocatechin 3-gallate intake as exposure factors.

All the models wereadjusted for age, gender, ethnicity, education level, marital status, poverty income ratio, body mass index, smoke, alcohol drinking status, and history of diabetes or hypertension.

WQS, weighted quantile sum; CI, confidence interval; OR, odds ratio.

**Figure 2:**
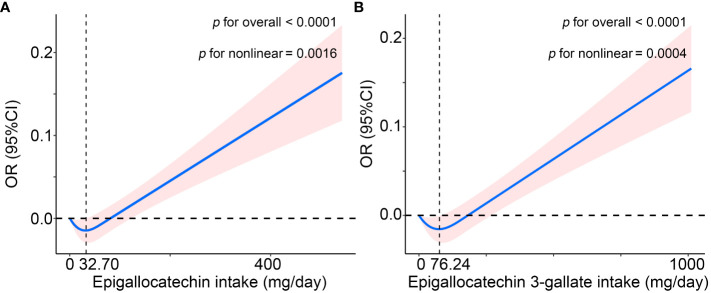
The non-linear association of osteoarthritis with **(A)** Epigallocatechin intake and **(B)** Epigallocatechin 3-gallate intake in the US (NHANES 2007-2010 and 2017-2018), using the restricted cubic spline (RCS) regression analysis. The solid red line is the OR value and the shadowed area is the corresponding 95% CI. The model was adjusted for age, gender, ethnicity, education level, marital status, poverty income ratio, body mass index, serum cotinine, alcohol drinking status, and history of diabetes or hypertension. CI, confidence interval; OR, odds ratio.

As shown in **Figure 1**, in the WQS regression model containing all catechins, the highest contribution to the WQS index was made by epigallocatechin (40%), and in the WQS regression model consisting of intakes of epigallocatechin and epigallocatechin 3-gallate, the highest contribution to the WQS index was still made by epigallocatechin (98.5%). Moreover, we further explored the associations with the regression coefficients assumed to be negative in the two WQS models. Unsurprisingly, the weight of Epigallocatechin in the models was zero and neither model is statistically significant (**Figure S1** and **Table S3**). Furthermore, the restricted cubic spline (RCS) modes adjusted for all confounders demonstrated a nonlinear and J-shaped association between the intakes of epigallocatechin (P for non-linearity = 0.0016) and Epigallocatechin 3-gallate (P for non-linearity = 0.0004) and risk of OA (**Figure 2**). The risk of OA reached a nadir when epigallocatechin at approximately 32.70 mg/day and epigallocatechin 3-gallate at approximately 76.24 mg/day, followed by a gradual increase in OR with increasing daily intake.

### Subgroup analysis

In the subgroup analyses, we stratified all covariates and used multifactorial logistic regression models adjusted for all confounders except the variables themselves to analyze the association between daily dietary epigallocatechin and epigallocatechin 3-gallate intake and the prevalence of OA. In addition, a multiplicative interaction term was added to each model for testing potential interactions, and the results indicated no significant interactions between daily dietary intake of epigallocatechin and epigallocatechin 3-gallate and the stratification variables (**Tables S1, 2**).

### Interaction effect between physical activity and epigallocatechin intake on the prevalence of OA

**Figure 3:**
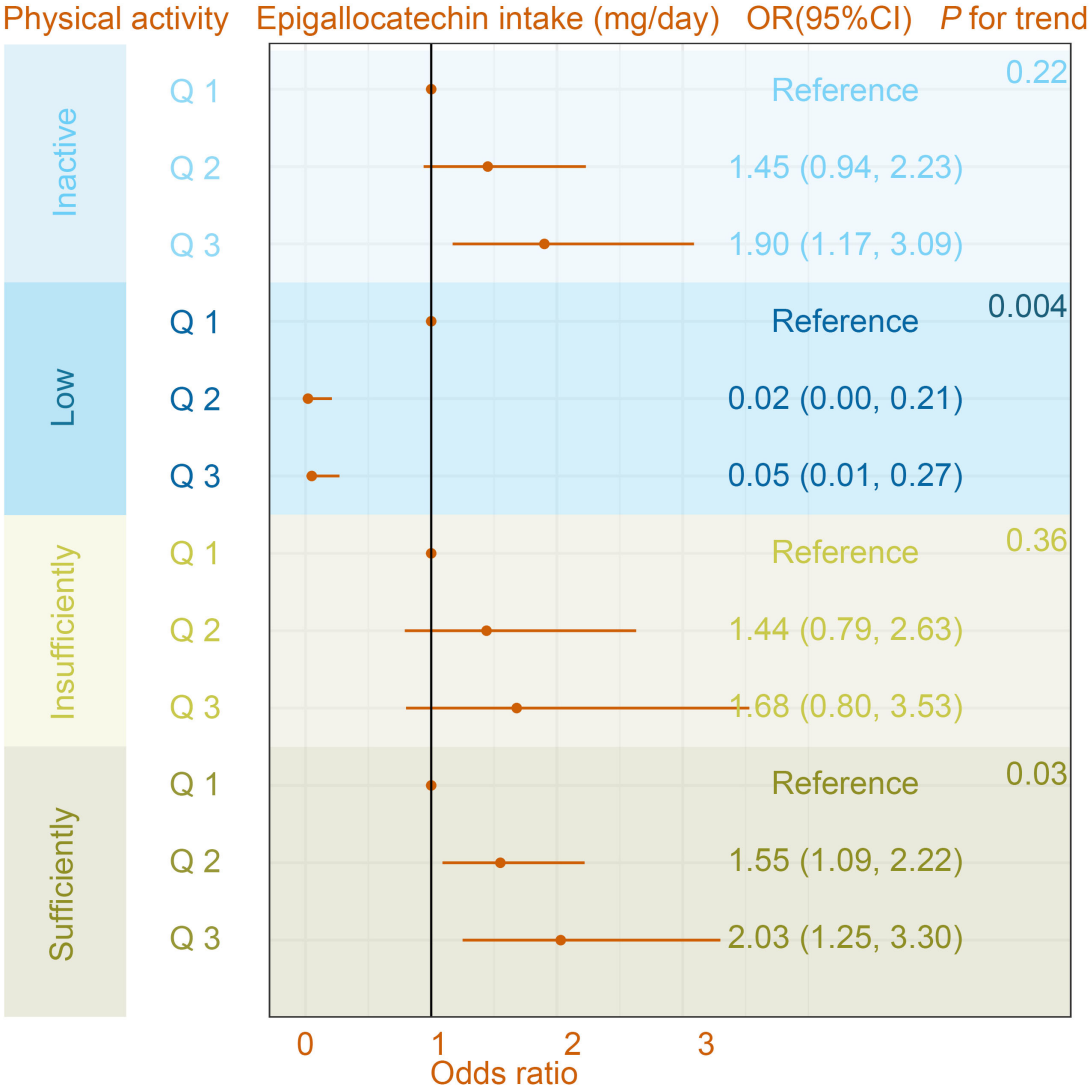
The association between osteoarthritis and Epigallocatechin intake in different physical activity subgroups. The model was adjusted for age, gender, ethnicity, education level, marital status, poverty income ratio, body mass index, serum cotinine, alcohol drinking status, and history of diabetes or hypertension. CI, confidence interval; OR, odds ratio.

In the interaction assay, as shown in **Table 4**, we found a significant moderating effect of PA in the relationship between epigallocatechin intake and the prevalence of OA (P for interaction = 0.03), whereas this interaction was not observed in the relationship between epigallocatechin 3-gallate and OA prevalence (P for interaction = 0.58). Specifically, in the Low PA group, the results of the multivariate-adjusted logistic regression model showed ORs (95%CI) were 0.02 (0.00, 0.21) and 0.05 (0.01, 0.27) for Q 2 and 3, respectively, compared to the Q 1 group (**Figure 3**). While in the sufficiently PA group, with Q 1 group being the reference, the ORs (95%CI) were Q 2 1.55 (1.09, 2.22) and Q 3 2.03 (1.25, 3.30). In addition, the results of the trend test were statistically significant in the Low PA group (P for trend = 0.004) and sufficiently PA group (P for trend = 0.03).

**Table 4:** Interaction effect between physical activity and Epigallocatechin or Epigallocatechin 3-gallate intake on the risk of osteoarthritis in the US (NHANES 2007-2010 and 2017-2018).

Variable	Adjusted OR (95% CI)			*p* for trend	*p* for interaction
	Q 1	Q 2	Q 3		
**Epigallocatechin (mg/day)**					0.03
Inactive	Reference	1.45 (0.94, 2.23)	1.90 (1.17, 3.09)	0.22	
Low	Reference	0.02 (0.00, 0.21)	0.05 (0.01, 0.27)	0.004	
Insufficiently	Reference	1.44 (0.79, 2.63)	1.68 (0.80, 3.53)	0.36	
Sufficiently	Reference	1.55 (1.09, 2.22)	2.03 (1.25, 3.30)	0.03	
**Epigallocatechin 3-gallate (mg/day)**	Reference				0.58
Inactive	Reference	1.46 (0.81, 2.66)	1.57 (1.02, 2.42)	0.07	
Low	Reference	2.31 (0.18, 29.41)	1.69 (0.58, 4.92)	0.66	
Insufficiently	Reference	0.66 (0.25, 1.75)	1.07 (0.71, 1.62)	0.38	
Sufficiently	Reference	1.49 (1.01, 2.21)	1.43 (1.02, 1.99)	0.08	

The model was adjusted for age, gender, ethnicity, education level, marital status, poverty income ratio, body mass index, smoke, alcohol drinking status, and history of diabetes or hypertension.

CI, confidence interval; OR, odds ratio.

## Discussion

In this study, the relationship between dietary catechins intake and the prevalence of OA was investigated for the first time, using the study cohort of 10039 participants from the NHANES database, including both OA and non-OA individuals. Our results suggest that a J-shaped nonlinearly correlation between the intakes of epigallocatechin and epigallocatechin 3-gallate with the risk of OA, in which PA played a significant moderating effect.

Most studies have found that catechins have a positive effect on OA treatment. The mechanisms are not fully understood, but several possible mechanisms have been suggested. Catechins could up-regulate the expression of nuclear factor erythrocyte 2-related factor 2 (Nrf2), oxygenase 1 (HO-1), NADPH quinone oxidoreductase 1 (NQO1), and other antioxidant enzymes, and improve the oxidative stress-induced chondrocyte dysfunction (26). Moreover, catechins can effectively clear excessive ROS in cells, significantly reduce the expression of pro-inflammatory cytokines, reduce the expression of M1-type macrophages, and show an excellent promotion effect on the transformation of macrophages to M2 phenotype (27, 28). However, the relationship between catechins intake and the risk of OA has not been evaluated in any study. In this research, we explored the associations between six catechins subclasses and the prevalence of OA, with multifactorial logistic regression and WQS regression model, and found that excessive intake of epigallocatechin and epigallocatechin 3-gallate increases the risk of OA in the general US population.

Several experimental animal studies and epidemiological studies have shown that tea polyphenols have dose-dependent toxicology and low and medium doses (0.01-0.25%) of tea polyphenols show beneficial effects in the large intestine, liver, and kidney (29). Conversely, a high dietary dose (0.5-1%) of GTP reduced the expression of antioxidant enzymes and heat shock protein (HSP), leading to the worsening of colitis and colorectal cancer in mice, and also causing liver and kidney dysfunction (30). In addition, there have been case reports that excessive consumption of tea extract can cause liver damage (31, 32). It has been suggested that this may be related to the properties of tea polyphenols. Mechanistically, studies have found that tea polyphenols can produce reactive oxygen species (ROS) through auto-oxidation. Low and medium doses of tea polyphenols produce low levels of ROS, which can activate Nrf2 to reduce oxidative stress, while high doses of tea polyphenols produce high levels of ROS, leading to apoptosis and tissue damage (33–35). All the above research evidence suggests that the daily intake of dietary catechins should take into account the complementary and toxicological effects of dose relationships. Noteworthy, our findings suggest that although catechins have some adjunctive therapeutic effects in the OA population, gallocatechin intake greater than 32.70 mg/d or gallocatechin 3-gallate intake greater than 76.24 mg/d significantly increased the risk of OA in the general population.

Interestingly, in the present study, using the WQS regression model, we first explored the mixed effect of intake of all catechins on the prevalence of OA and the results showed that this model was not associated with the prevalence of OA. Subsequently, we focused on the mixed effect of intake of epigallocatechin and epigallocatechin 3-gallate, and unsurprisingly, there was significance between the model and the prevalence of OA. Therefore, the results of the WQS regression model showed that the overall effect of all six catechins subclasses or epigallocatechin and epigallocatechin 3-gallate mainly resulted from Epigallocatechin suggesting that Epigallocatechin intakes are more important for studying the prevalence of OA in the general U.S. population. Not alone, we found that there was no significant interaction effect in the intake of epigallocatechin and OA prevalence in age, gender, and ethnicity subgroups, while a significant interaction effect was found in the PA subgroup. According to the 2018 Physical Activity (PA) Guidelines from the U.S. Department of Health and Human Services (DHHS), maintaining a moderate intensity of physical activity each week can reduce OA risk and also have a positive effect on OA recovery (18, 36). Many reports have shown that high-intensity exercise itself is easy to cause joint strain, which has shown that high-intensity exercise is an increased risk of OA (19, 37, 38). Similarly, our study found that intake of epigallocatechin hardly affected the protective effect of low PA on the risk of OA in the Low PA group. However, in the Sufficiently PA group, the prevalence of OA was significantly higher in the epigallocatechin Q 3 group compared to the Q 1 group, and OA prevalence increased with the higher daily intake of epigallocatechin.

This study is a relatively large population study using three complementary methods to reveal the relationship between dietary catechin intake and the prevalence of OA in American adults. Under the premise that dietary catechins are now recommended as natural health products in daily life, we first found that excessive daily catechins intake will lead to an increase in the risk of OA. However, further large-scale prospective studies and clinical trials are needed to confirm our findings and their underlying mechanisms. In addition, there are some limitations to our study. First, we used data from a cross-sectional survey. The assessment of dietary catechin intake in this study can only reflect current intake status, but OA is a long-term developing disease, which may have biased our results. Second, dietary catechins intake data were collected through a 24-hour dietary recall survey, which could lead to recall bias. Third, our analysis was unable to conclude a causal relationship between dietary catechin intake and OA. Fourth, our population inclusion is limited by the NHANES database. It is unclear whether the relationship between dietary catechin intake and OA applies to other populations.

## Conclusion

In summary, the results of this study suggested that epigallocatechin intake greater than 32.70 mg/d or epigallocatechin 3-gallate intake greater than 76.24 mg/d significantly increases the risk of OA in the general US population. In addition, PA showed a significant moderating effect on the relationship between epigallocatechin intake and the prevalence of OA.

## Data availability statement

The original contributions presented in the study are included in the article/**Supplementary Material**. Further inquiries can be directed to the corresponding authors.

## Author contributions

YF: Conceptualization. LL: Conceptualization. JG: Writing – original draft. FW: Writing – review & editing. ZZ: Writing – review & editing. YZ: Project administration.
