# Supplementary material for: J-shaped association of dietary catechins intake with the prevalence of osteoarthritis and moderating effect of physical activity: an American population-based cohort study
Source: Front Immunol. 2024 Jan 8;14:1287856. doi: 10.3389/fimmu.2023.1287856 (PMC10801035; doi:10.3389/fimmu.2023.1287856)
Supplement: Supplementary file 1 [file DataSheet_1.docx]

§Table S1. The association between the Epigallocatechin intake and osteoarthritis among different subgroups in the US.

| **Characteristic** | **Epigallocatechin (mg/day)**  **Adjusted OR (95% CI)** | | | ***p* for trend** | ***p* for interaction** |
| --- | --- | --- | --- | --- | --- |
|  | **Q 1** | **Q 2** | **Q 3** |  |  |
| **Age (years)** |  |  |  |  | 0.28 |
| 20-59 | Reference | 1.67 (1.09, 2.56) | 2.18 (1.40, 3.40) | 0.01 |  |
| > = 60 | Reference | 1.04 (0.76, 1.43) | 1.28 (0.86, 1.90) | 0.2 |  |
| **Gender** |  |  |  |  | 0.93 |
| Female | Reference | 1.40 (1.01, 1.95) | 1.86 (1.22, 2.84) | 0.01 |  |
| Male | Reference | 1.35 (0.83, 2.19) | 1.59 (0.96, 2.63) | 0.21 |  |
| **Ethnicity** |  |  |  |  | 0.63 |
| Non-Hispanic Black | Reference | 0.96 (0.62, 1.49) | 1.20 (0.66, 2.21) | 0.46 |  |
| Non-Hispanic White | Reference | 1.43 (1.07, 1.91) | 1.81 (1.25, 2.63) | 0.03 |  |
| Mexican American | Reference | 0.95 (0.38, 2.41) | 1.17 (0.55, 2.49) | 0.56 |  |
| Other Hispanic | Reference | 0.96 (0.54, 1.70) | 1.19 (0.38, 3.75) | 0.7 |  |
| Other Race | Reference | 2.69 (1.13, 6.41) | 4.09 (1.73, 9.65) | 0.02 |  |
| **Education level** |  |  |  |  | 0.58 |
| Less than high school | Reference | 1.10 (0.63, 1.92) | 1.45 (0.85, 2.49) | 0.14 |  |
| High school or equivalent | Reference | 1.13 (0.70, 1.83) | 1.83 (0.91, 3.68) | 0.07 |  |
| College or above | Reference | 1.66 (1.08, 2.55) | 1.91 (1.29, 2.84) | 0.03 |  |
| **Marital status** |  |  |  |  | 0.73 |
| Married/cohabiting | Reference | 1.22 (0.90, 1.67) | 1.54 (1.11, 2.14) | 0.06 |  |
| Widowed/divorced/separated | Reference | 1.53 (1.03, 2.27) | 2.12 (1.35, 3.34) | 0.02 |  |
| Never married | Reference | 1.59 (0.69, 3.65) | 2.03 (0.89, 4.63) | 0.13 |  |
| **Poverty income ratio** |  |  |  |  | 0.92 |
| < 1.3 | Reference | 1.61 (0.88, 2.94) | 1.89 (1.03, 3.47) | 0.09 |  |
| 1.3-3.49 | Reference | 1.28 (0.84, 1.97) | 1.56 (1.03, 2.36) | 0.1 |  |
| > = 3.5 | Reference | 1.35 (0.88, 2.09) | 1.82 (1.14, 2.91) | 0.03 |  |
| **Body mass index (kg/m2)** |  |  |  |  | 0.35 |
| < 25 | Reference | 1.80 (1.07, 3.03) | 2.00 (1.29, 3.12) | 0.12 |  |
| > = 25-30 | Reference | 1.26 (0.96, 1.65) | 1.66 (1.14, 2.42) | 0.03 |  |
| **Smoke (ng/ml)** |  |  |  |  | 0.87 |
| no | Reference | 1.49 (0.98, 2.27) | 1.85 (1.18, 2.88) | 0.04 |  |
| yes | Reference | 1.32 (0.91, 1.94) | 1.71 (1.18, 2.46) | 0.03 |  |
| **Alcohol drinking** |  |  |  |  | 0.96 |
| Non-drinker | Reference | 1.08 (0.53, 2.21) | 1.69 (0.78, 3.66) | 0.09 |  |
| Drinker | Reference | 1.43 (1.09, 1.87) | 1.82 (1.31, 2.52) | 0.01 |  |
| **Diabetes** |  |  |  |  | 0.05 |
| no | Reference | 1.47 (1.07, 2.02) | 1.94 (1.34, 2.80) | 0.01 |  |
| yes | Reference | 0.97 (0.54, 1.74) | 1.01 (0.54, 1.87) | 0.91 |  |
| **Hypertension** |  |  |  |  | 0.68 |
| no | Reference | 1.14 (0.74, 1.75) | 1.64 (1.04, 2.61) | 0.03 |  |
| yes | Reference | 1.64 (1.20, 2.23) | 1.86 (1.25, 2.78) | 0.06 |  |

The model was adjusted for all variables except the variables themselves.

Abbreviation: CI, confidence interval; OR, odds ratio.

Table S2. The association between the Epigallocatechin 3-gallate intake and osteoarthritis among different subgroups in the US.

| **Characteristic** | **Epigallocatechin 3**-**gallate (mg/day)**  **Adjusted OR (95% CI)** | | | ***p* for trend** | ***p* for interaction** |
| --- | --- | --- | --- | --- | --- |
|  | **Q 1** | **Q 2** | **Q 3** |  |  |
| **Age (years)** |  |  |  |  | 0.46 |
| 20-59 | Reference | 1.34 (0.86, 2.07) | 1.57 (1.14, 2.16) | 0.01 |  |
| > = 60 | Reference | 1.25 (0.79, 1.99) | 1.21 (0.87, 1.68) | 0.41 |  |
| **Gender** |  |  |  |  | 0.05 |
| Female | Reference | 1.18 (0.77, 1.82) | 1.58 (1.10, 2.27) | 0.01 |  |
| Male | Reference | 1.78 (1.12, 2.82) | 1.10 (0.79, 1.55) | 0.89 |  |
| **Ethnicity** |  |  |  |  | 0.46 |
| Non-Hispanic Black | Reference | 0.90 (0.49, 1.66) | 1.33 (0.76, 2.32) | 0.27 |  |
| Non-Hispanic White | Reference | 1.35 (0.89, 2.06) | 1.33 (0.99, 1.79) | 0.1 |  |
| Mexican American | Reference | 0.86 (0.44, 1.66) | 0.94 (0.49, 1.80) | 0.97 |  |
| Other Hispanic | Reference | 1.16 (0.42, 3.17) | 1.22 (0.48, 3.10) | 0.72 |  |
| Other Race | Reference | 1.77 (0.50, 6.29) | 3.54 (1.57, 7.95) | 0.003 |  |
| **Education level** |  |  |  |  | 0.14 |
| Less than high school | Reference | 0.45 (0.23, 0.89) | 1.23 (0.80, 1.91) | 0.08 |  |
| High school or equivalent | Reference | 1.57 (0.82, 2.99) | 1.47 (0.82, 2.65) | 0.28 |  |
| College or above | Reference | 1.47 (0.95, 2.28) | 1.40 (1.08, 1.82) | 0.06 |  |
| **Marital status** |  |  |  |  | 0.12 |
| Married/cohabiting | Reference | 1.26 (0.82, 1.93) | 1.21 (0.89, 1.65) | 0.34 |  |
| Widowed/divorced/separated | Reference | 1.64 (0.94, 2.86) | 1.85 (1.24, 2.74) | 0.004 |  |
| Never married | Reference | 0.37 (0.12, 1.12) | 1.67 (0.86, 3.25) | 0.07 |  |
| **Poverty income ratio** |  |  |  |  | 0.95 |
| < 1.3 | Reference | 0.99 (0.43, 2.25) | 1.29 (0.79, 2.12) | 0.21 |  |
| 1.3-3.49 | Reference | 1.39 (0.84, 2.30) | 1.40 (1.03, 1.92) | 0.05 |  |
| > = 3.5 | Reference | 1.30 (0.76, 2.21) | 1.39 (0.96, 2.01) | 0.13 |  |
| **Body mass index (kg/m2)** |  |  |  |  | 0.75 |
| < 25 | Reference | 1.49 (0.77, 2.90) | 1.57 (1.01, 2.44) | 0.1 |  |
| > = 25-30 | Reference | 1.27 (0.81, 1.97) | 1.33 (0.98, 1.80) | 0.06 |  |
| **Smoke (ng/ml)** |  |  |  |  | 0.74 |
| no | Reference | 1.28 (0.76, 2.13) | 1.47 (0.98, 2.20) | 0.05 |  |
| yes | Reference | 1.37 (0.86, 2.20) | 1.31 (0.94, 1.82) | 0.17 |  |
| **Alcohol drinking** |  |  |  |  | 0.81 |
| Non-drinker | Reference | 1.39 (0.61, 3.17) | 1.53 (0.81, 2.90) | 0.3 |  |
| Drinker | Reference | 1.30 (0.90, 1.87) | 1.39 (1.07, 1.81) | 0.02 |  |
| **Diabetes** |  |  |  |  | 0.13 |
| no | Reference | 1.34 (0.93, 1.92) | 1.47 (1.14, 1.90) | 0.01 |  |
| yes | Reference | 1.14 (0.54, 2.39) | 0.92 (0.54, 1.55) | 0.64 |  |
| **Hypertension** |  |  |  |  | 0.28 |
| no | Reference | 0.99 (0.59, 1.66) | 1.40 (0.97, 2.03) | 0.05 |  |
| yes | Reference | 1.63 (1.02, 2.61) | 1.37 (1.01, 1.86) | 0.13 |  |

The model was adjusted for all variables except the variables themselves.

Abbreviation: CI, confidence interval; OR, odds ratio.

Table S3. When assuming that the regression coefficient was negative, the association between the catechins intake and osteoarthritis in the US by WQS analysis.

|  | **Adjusted OR (95% CI)** | | ***p*-value** |
| --- | --- | --- | --- |
| **WQS (all catechins)** | | 1.02 (0.96, 1.08) | 0.50 |
| **WQS (****Epigallocatechin and Epigallocatechin 3-gallate)** | | 1.02 (0.96, 1.09) | 0.47 |

All the models were adjusted for age, gender, ethnicity, education level, marital status, poverty income ratio, body mass index, smoke, alcohol drinking status, and history of diabetes or hypertension.

Abbreviation: WQS, weighted quantile sum; CI, confidence interval; OR, odds ratio.


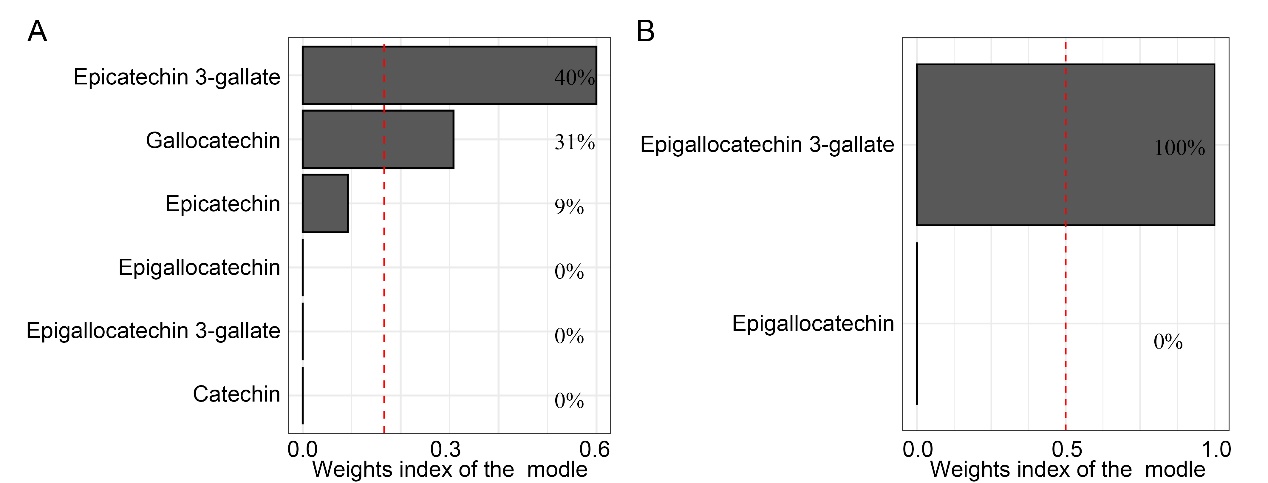


**Fig.S1.** The weighted quantile sum (WQS) regression model index weights for the WQS regression model included (A) all catechins and (B) only Epigallocatechin and Epigallocatechin 3-gallate when assuming that the regression coefficient was negative. The red dashed line indicates the inverse of the number of exposed variables in the model. The model was adjusted for age, gender, ethnicity, education level, marital status, poverty income ratio, body mass index, serum cotinine, alcohol drinking status, and history of diabetes or hypertension.
